# BRD4 Mediates Cadmium-Induced Oxidative Stress and Kidney Injury in Mice via Disruption of Redox Homeostasis

**DOI:** 10.3390/toxics13040258

**Published:** 2025-03-29

**Authors:** Jiaxin Chen, Guangling Guo, Xinyu Wang, Zifa Li, Tingru Ji, You Li, Hongwei Dong, Hao Zhang, Mingzhou Gao

**Affiliations:** 1 College of Traditional Chinese Medicine, Shandong University of Traditional Chinese Medicine, Jinan 250355, China; jnlwcjx@126.com (J.C.); 17661258518@163.com (G.G.); 2 Experimental Center, Shandong University of Traditional Chinese Medicine, Jinan 250355, China; wangxinyu0601@163.com (X.W.); zifa_0611@163.com (Z.L.); 3 College of Acupuncture-Moxibustion and Tuina, Shandong University of Traditional Chinese Medicine, Jinan 250355, China; jitingru_1204@163.com (T.J.); 15650097275@163.com (Y.L.); 4 School of Pharmacy, Shandong University of Traditional Chinese Medicine, Jinan 250355, China; donghongwei2012@163.com; 5 High-Level Key Disciplines of Traditional Chinese Medicine: Basic Theory of Traditional Chinese Medicine, National Administration of Traditional Chinese Medicine, Shandong University of Traditional Chinese Medicine, Jinan 250355, China; 6 Innovation Research Institute of Traditional Chinese Medicine, Shandong University of Traditional Chinese Medicine, Jinan 250355, China

**Keywords:** cadmium, BRD4, oxidative stress, kidney injury, toxicology

## Abstract

Cadmium (Cd) is a toxic heavy metal that threatens public health, with kidney injury being one of the common manifestations after Cd exposure. Oxidative stress plays a crucial role in Cd-induced kidney injury, arising from an imbalance between cellular oxidation and antioxidation processes. Bromodomain-containing protein 4 (BRD4) has been identified as a significant factor in the initiation and advancement of multiple diseases, primarily due to its regulatory role in oxidative stress. Nevertheless, the specific role of BRD4 in Cd-induced kidney oxidative injury remains poorly understood. The present study demonstrates that BRD4 is activated in the kidney after Cd exposure, while JQ1 (a BRD4 inhibitor) treatment inhibits Cd-induced oxidative stress and kidney injury. Subsequently, we investigate the mechanisms by which Cd regulates oxidative stress both in vivo and in vitro. The results indicate that JQ1 treatment reduces the expression levels of NADPH oxidase 4 (Nox4), thereby alleviating mitochondrial damage and reducing reactive oxygen species (ROS) generation. Furthermore, JQ1 treatment facilitates nuclear translocation levels of Nuclear factor erythroid-derived 2-like 2 (Nrf2), thereby enhancing the antioxidant defense system in the kidney after Cd exposure. In conclusion, this study reveals that BRD4 is significantly involved in the process of Cd-induced oxidative damage in the kidney, while inhibiting BRD4 is observed to attenuate ROS generation by regulating Nox4 and enhance ROS scavenging by regulating Nrf2, which, in turn, suppresses the oxidative stress level in the kidney after Cd exposure. These findings suggest that targeting BRD4 may represent an effective strategy for the prevention and treatment of Cd-induced kidney diseases.

## 1. Introduction

Cadmium (Cd) is a widespread environmental pollutant and represents a significant risk to public health [1,2]. Apart from occupational exposure, the general population is mainly exposed to Cd through the consumption of Cd-contaminated water, food, and cigarettes [3]. Cd exposure has been linked to a range of adverse effects on multiple organs, including nephrotoxicity, hepatotoxicity, testicular damage, and lung cancer [4,5,6]. Given the growing recognition of Cd pollution as a significant public health concern, the International Agency for Research on Cancer (IARC) has classified it as a type I carcinogen, which underscores the urgent need to elucidate the toxic mechanisms of Cd and identify efficacious protective agents [7].

Oxidative stress is a disruption of the intracellular balance between oxidants and antioxidants, resulting in the overproduction of reactive oxygen species (ROS). This phenomenon is considered the primary molecular mechanism underlying Cd-induced cell toxicity [8,9]. Oxidative stress contributes significantly to various disease mechanisms induced by Cd exposure, such as cancer, neurological disorders, and others [10,11]. The extent of oxidative stress is influenced by the equilibrium between the generation of ROS and their elimination, with numerous regulators identified as participating in this regulatory mechanism [12]. Mitochondria are the main site of ROS production, while NADPH oxidase 4 (Nox4) has been reported to mediate mitochondrial dysfunction to aggravate ROS accumulation [13]. Nuclear factor erythroid-derived 2-like 2 (Nrf2) is widely recognized as a critical system for antioxidant defense in the body. This pathway is essential for enhancing endogenous antioxidant capacity, primarily by regulating the transcription levels of antioxidant enzymes and phase II detoxification enzymes [14]. After Cd exposure, the altered expression of oxidative stress regulators disrupts the balance between the intracellular oxidative and antioxidant defense systems, inducing oxidative stress, and then leads to tissue injury [15].

In recent years, epigenetic regulation of cell biological processes has gradually become an emerging strategy [16,17]. Bromodomain and extra-terminal (BET) family proteins contain two structural N-terminal bromodomains (BD1 and BD2) [18]. Among the BET family proteins, BRD4 is the most widely studied [19]. By recognizing and then binding acetylation-modified histones through its bromo-structural domains, BRD4 functions to decipher the epigenetic code [20]. JQ1 is a novel small molecule that selectively targets and competes with BRD4, binding to all bromo-structural domains of the BET family proteins, thus releasing BRD4 from chromatin and inhibiting the transcriptional regulation of BRD4 [21]. Studies have shown that BRD4 is crucial in the regulation of oxidative stress and kidney injury. Zhou et al. found that JQ1 inhibited the transcriptional activity of Nox4, leading to a reduction in Nox4-mediated ROS generation and fibrotic gene expression [22]. Additionally, Gong et al. reported that BRD4 is implicated in Cd-induced acute kidney injury, inhibiting autophagy and regulating oxidative stress [23].

This study explored the role of BRD4 in Cd-induced oxidative stress of the kidney in vivo and in vitro. The results showed that Cd exposure activates the epigenetic regulator BRD4, while JQ1 treatment decreases Nox4-regulated ROS generation and increases Nrf2-regulated antioxidant levels, thereby reducing oxidative damage and exerting a protective effect. These findings corroborate the hypothesis of a new role of BRD4 in Cd-induced renal injury and provide evidence that may inform the prevention and treatment of Cd-induced renal diseases.

## 2. Materials and Methods

### 2.1. Reagents and Equipment

Cadmium chloride (CdCl_2_, anhydrous, Cat#: 439800) and 2′,7′-dichlorofuorescein diacetate (DCFH-DA, Cat#: D6883) were sourced from Sigma-Aldrich (St. Louis, MO, USA). The enhanced cell counting kit-8 (CCK-8, Cat#: C0042), Nuclear Protein Extraction Kit (Cat#: P0028), Malondialdehyde (MDA) assay kit (Cat#: S0131S), Hematoxylin and Eosin Staining Kit (Cat#: C0105S), DAPI Staining Solution (Cat#: C1005), and enhanced mitochondrial membrane potential assay kit with JC-1 (Cat#: C2003S) were obtained from Beyotime Biotechnology (Nantong, Jiangsu, China). The compound (+)-JQ-1 (JQ1, Cat#: HY-13030) was sourced from MedChemExpress (Monmouth Junction, NJ, USA). The primary antibodies utilized in this study were as follows: the β-actin antibody (A5441) was obtained from Sigma (St. Louis, MO, USA); the BRD4 antibody (ab128874) was purchased from Abcam (Cambridge, UK); and antibodies against Histone H3 (17168-1-AP), Nrf2 (16396-1-AP), and Nox4 (14347-1-AP), along with all secondary antibodies, were procured from Proteintech Group (Wuhan, Hubei, China).

A real-time fluorescence quantitative PCR instrument (Roche, Basel, Switzerland), Chemidoc XRS system (Bio-Rad, Marnes-La-Coquette, France), CyAn ADP 7 flow cytometer (Beckman Coulter, Brea, CA, USA), and TCS SP8 confocal microscope (Leica, Wetzlar, Germany) were used in the following analyses.

### 2.2. Animals and Experiments

Twenty-four C57BL/6 male mice (6 weeks old, 18–20 g) were purchased from Pengyue Experimental Animal Breeding Co., Ltd. (Jinan, Shandong, China). The mice were maintained in a stable environment with controlled temperature and light (22 ± 2 °C, 12 h light/dark cycle). The experimental procedures were in accordance with European Community Council Directive 2010/63/EU on animal experimentation and approved by the Shandong University of Traditional Chinese Medicine Animal Care and Use Committee (SDUTCM20241015502). Mice were randomly assigned to 4 groups: the Cont group (normal saline, 7 days, injected intraperitoneally), Cd group (CdCl_2_, 1 mg/kg/day for 7 days, injected intraperitoneally), Cd+JQ1 group (JQ1, 30 mg/kg/day and CdCl_2_, 1 mg/kg/day for 7 days, injected intraperitoneally), and JQ1 group (JQ1, 30 mg/kg/day, 7 days, injected intraperitoneally). The concentrations of Cd and JQ1 were determined with reference to previous studies [23,24]. During the experimental period, the mice were provided with unrestricted access to food and water.

Following the treatment period, the rats were euthanized by cervical dislocation under deep anesthesia after a 12 h fast. The kidney tissues were meticulously dissected, and a portion of the kidney tissues was promptly stored at −80 °C for subsequent Western blot and qPCR analysis. The remaining tissues were rapidly fixed in 4% paraformaldehyde for subsequent morphological observation.

### 2.3. Cell Culture

Cultured renal proximal tubule cells (TKPTS cells) were purchased from ATCC (CRL-3361) and were maintained in DMEM supplemented with 10% FBS. All cell culture and handling procedures took place in a sterile environment. The cells were placed in suitable culture plates based on the experimental design. When the cells had grown to 80%, the appropriate drug treatments were performed: (1) TKPTS cells were treated with 0, 2.5, 5, or 10 μM Cd for 12 h to perform the subsequent assays. (2) TKPTS cells were treated with 5 μM Cd for 12 h and 0.5 μM JQ1 for another 12 h to analyze the chosen indicators. The concentrations of Cd and JQ1 were determined with reference to previous studies [23].

### 2.4. qPCR Analysis

RNAiso was used to extract total RNA and then reverse-transcribed to cDNA, and the LightCycler^®^ 96 RT-PCR system (Roche Diagnostics, Basel, Switzerland) was used to analyze the transcription levels. The qPCR process consisted of pre-denaturation at 95 °C for 3 min, followed by 30 cycles of denaturation at 95 °C for 30 s, low-temperature annealing at 55 °C for 45 s, and extension at 72 °C for 30 s. The primer sequences are listed in Table S1.

### 2.5. Western Blot Analysis

Proteins were isolated from TKPTS cells and kidney tissues using RIPA lysis buffer supplemented with a mixture of protease inhibitors. For the analysis, 20 µg of each sample was loaded. The samples underwent sodium dodecyl sulfate–polyacrylamide gel electrophoresis (SDS-PAGE) and were subsequently transferred to polyvinylidene fluoride (PVDF) membranes. Following the transfer, the membranes were blocked (5% skim milk, 90 min), then incubated with primary antibodies against BRD4, Nox4, Nrf2, Histone H3 (all diluted 1:1000), and β-actin (diluted 1:5000). Then, they were incubated with secondary antibodies (diluted 1:10,000) for 50 min and visualized using the Chemidoc XRS system (Bio-Rad, Marnes-La-Coquette, France).

### 2.6. Histopathological Observation

Kidney tissue was excised, bisected, and fixed in a 4% paraformaldehyde solution. Following alcohol gradient dehydration, toluene transparency, paraffin embedding, sectioning, and hematoxylin–eosin (H&E) staining, the kidney tissue was observed and recorded under a light microscope to ascertain the pathological changes.

### 2.7. MDA and ROS Assay

The levels of MDA in both cellular and kidney tissue samples were measured using an MDA assay kit specifically designed for such quantification. To assess the presence of intracellular ROS, the DCFH-DA staining technique was employed. After TKPTS cells were subjected to the specified treatments, the cells were treated with DCFH-DA. The resulting DCF fluorescence, indicative of ROS levels, was analyzed using a CyAn ADP 7 flow cytometer (Beckman Coulter, Brea, CA, USA). Additionally, the quantification of ROS levels in kidney tissues was carried out using a method detailed in [25].

### 2.8. Immunofluorescence (IF) Staining

TKPTS cells were fixed, then incubated with primary antibody for 12 h at 4 °C, then incubated with a fluorescent secondary antibody for 50 min and DAPI for 5 min. Subsequently, the stained proteins were observed under a Leica confocal microscope.

### 2.9. Mitochondrial Membrane Potential Analysis

The mitochondrial membrane potential was quantified using the JC-1 fluorescence assay. TKPTS cells were incubated with JC-1 for 30 min and then analyzed with a CyAn ADP 7 flow cytometer.

### 2.10. Adenosine Triphosphate (ATP) Content Determination

The ATP content was analyzed using commercial kits following the manufacturer’s provided instructions. Initially, cells underwent homogenization using a lysis buffer to ensure effective cell disruption and release of cellular contents. Following this step, the resulting lysate was subjected to centrifugation for 10 min at a speed of 12,000 rpm. The protein concentration of the collected supernatant was then determined utilizing a protein assay kit, ensuring accurate quantification of the protein levels present in the sample. Finally, to assess ATP levels, the samples were mixed with the ATP test reagent and incubated for 5 min, after which the absorbance value was measured to evaluate the ATP content in the samples.

### 2.11. Mito-Tracker Red (MTR)

TKPTS cells were combined with MTR staining solution and incubated for 30 min. Subsequently, the stained mitochondria were observed under a Leica confocal microscope.

### 2.12. Statistical Analysis

All data are presented as the mean ± SD. Experimental groups were compared using a two-tailed unpaired *t*-test or one-way ANOVA with Tukey or Dunnett post hoc tests, conducted with SPSS version 22.0 (SPSS Inc., Chicago, IL, USA).

## 3. Results

### 3.1. Cd Exposure Activates BRD4 in the Kidney

**Figure 1:**
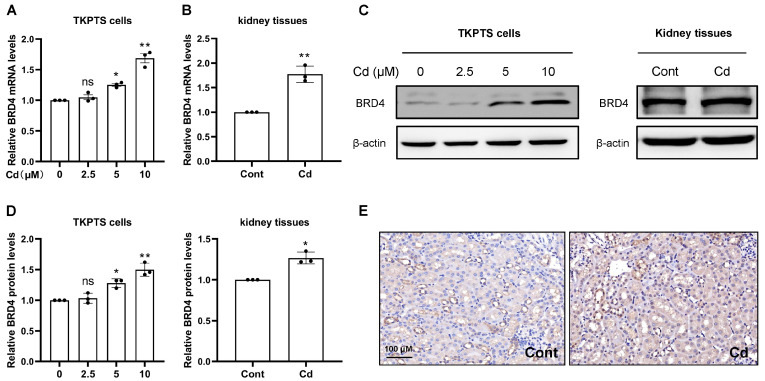
Cadmium (Cd) activates renal BRD4 in vitro and in vivo. (**A**) TKPTS cells underwent treatment with various concentrations of Cd (0, 2.5, 5, 10 μM) for 12 h to evaluate the transcription levels of BRD4. (**B**) Mice received daily intraperitoneal injections of CdCl_2_ for a duration of 7 consecutive days to assess BRD4 transcription levels in kidney tissues. (**C**) The levels of BRD4 expression in both TKPTS cells and kidney tissues were measured using Western blot analysis. (**D**) An analysis of the densitometry for BRD4 protein levels was performed. (**E**) Representative images showcasing BRD4 immunohistochemical staining are provided (scale bar = 100 µm). Mean ± SD, n = 3. ns not significant, * p < 0.05, ** p < 0.01 compared to the control group.

To explore the relationship between BRD4 and Cd-induced kidney injury, the activities of BRD4 were detected in vivo and in vitro. Firstly, the data presented in Figure 1A,B indicate that exposure to Cd elevated the transcription levels of BRD4 in a dose-dependent fashion. Additionally, the findings illustrated in Figure 1C,D reveal an increase in the expression levels of BRD4 after Cd exposure. Subsequently, the results of immunohistochemical (IHC) staining demonstrate that Cd exposure augmented the expression levels of BRD4 in kidney tissues (Figure 1E). Collectively, these findings imply that BRD4 might play a role in the context of Cd-induced kidney injury.

### 3.2. Inhibition of BRD4 Mitigates Kidney Injury Caused by Cd Exposure

**Figure 2:**
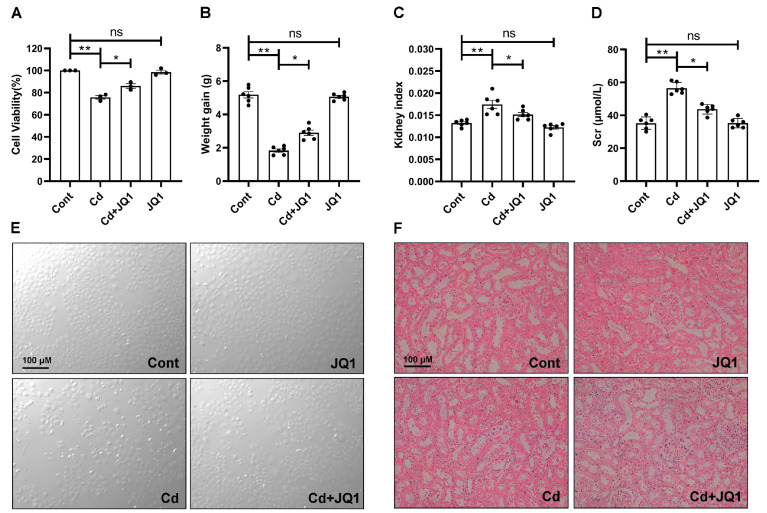
The inhibition of BRD4 alleviates Cd-induced kidney injury. (**A**,**E**) TKPTS cells were exposed to 5 μM Cd and/or 0.5μM JQ1 for 12 h, during which the CCK-8 assay (**A**) and a morphological assessment (**E**) were conducted to observe alterations in cellular damage. (**B**–**D**,**F**) Mice were treated with CdCl_2_ and/or JQ1, and their weight gain (**B**), kidney index (**C**), serum creatinine levels (**D**), and representative images from H&E staining (**F**) were employed to assess the extent of kidney injury. Mean ± SD, n = 3. ns not significant, * p < 0.05, ** p < 0.01.

Subsequently, the involvement of BRD4 in Cd-induced kidney injury was evaluated both in vivo and in vitro. As illustrated in Figure 2A, exposure to Cd decreased the viability of TKPTS cells, which was reversed by treatment with JQ1. Also, JQ1 treatment was observed to significantly mitigate Cd-induced cellular damage (Figure 2E). Additionally, the data in Figure 2B and C demonstrate that JQ1 treatment resumed the weight gain and decreased the kidney index in Cd-exposed mice. JQ1 treatment reduced the Cd-increased serum creatinine content (Figure 2D). Following this, H&E staining was utilized to evaluate the effect of JQ1 on renal histopathological damage induced by Cd. The results shown in Figure 2F revealed that Cd exposure caused the deterioration of tubular epithelial cells, while JQ1 treatment significantly minimized Cd-induced kidney injury. These findings support the idea that inhibiting BRD4 provides a protective effect against kidney injury triggered by Cd exposure.

### 3.3. Inhibition of BRD4 Attenuates Cd-Induced Oxidative Stress in Mice Kidney

**Figure 3:**
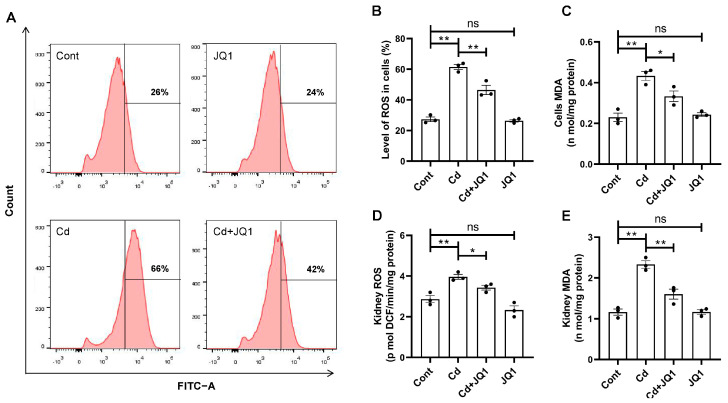
The inhibition of BRD4 alleviates Cd-mediated oxidative stress. Representative flow cytometry results (**A**), quantitative 2′,7′-dichlorofuorescein diacetate (DCFH-DA) results (**B**), and malondialdehyde (MDA) levels (**C**) were obtained to analyze the reactive oxygen species (ROS) levels in TKPTS cells. The ROS levels (**D**) and MDA levels (**E**) were detected to analyze the ROS levels in kidney tissues. Mean ± SD, n = 3. ns not significant, * p < 0.05, ** p < 0.01.

Oxidative stress represents a significant contributing factor to Cd-induced kidney injury in mice, while BRD4 has been shown to be essential in modulating oxidative stress across various pathological conditions. Next, this research investigated the role of BRD4 in governing oxidative stress in kidneys exposed to Cd. First, the results from flow cytometry assays indicated that the rise in intracellular ROS levels induced by Cd was significantly reduced by JQ1 in TKPTS cells (Figure 3A,B). MDA, an important indicator to evaluate the oxidative stress level, was found to increase following Cd exposure, while JQ1 treatment decreased Cd-increased MDA levels (Figure 3C). In addition, both flow cytometry and ELISA assays demonstrated that the increases in ROS and MDA levels caused by Cd were substantially diminished by JQ1 in kidney tissues (Figure 3D,E). These results indicate that BRD4 may be crucial in the development of Cd-induced kidney injury through its role in regulating oxidative stress.

### 3.4. JQ1 Treatment Inhibits Nox4-Mediated ROS Generation After Cd Exposure

**Figure 4:**
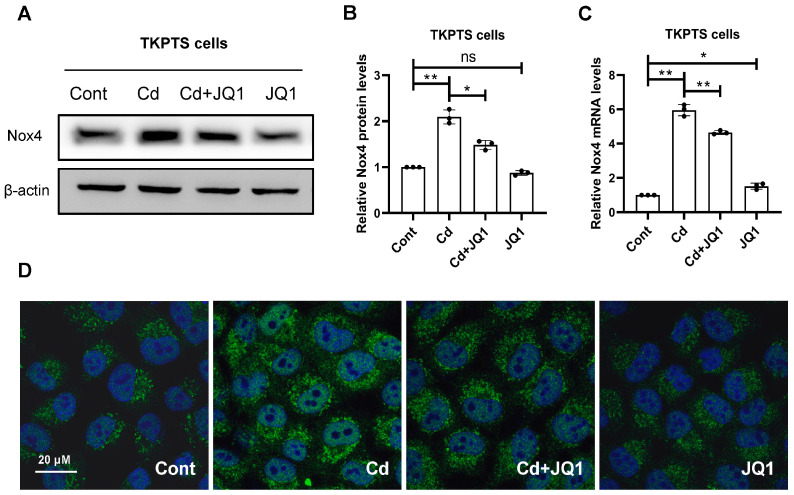
The inhibition of BRD4 decreases Cd-increased expression of Nox4. (**A**) The expression levels of Nox4 were detected by Western blot analysis. (**B**) An analysis of the densitometry for Nox4 protein levels was performed. (**C**) The transcription levels of Nox4 were assessed through qRT-PCR. (**D**) The expression levels of Nox4 were evaluated using IF staining. Mean ± SD, n = 3. ns not significant, * p < 0.05, ** p < 0.01.

**Figure 5:**
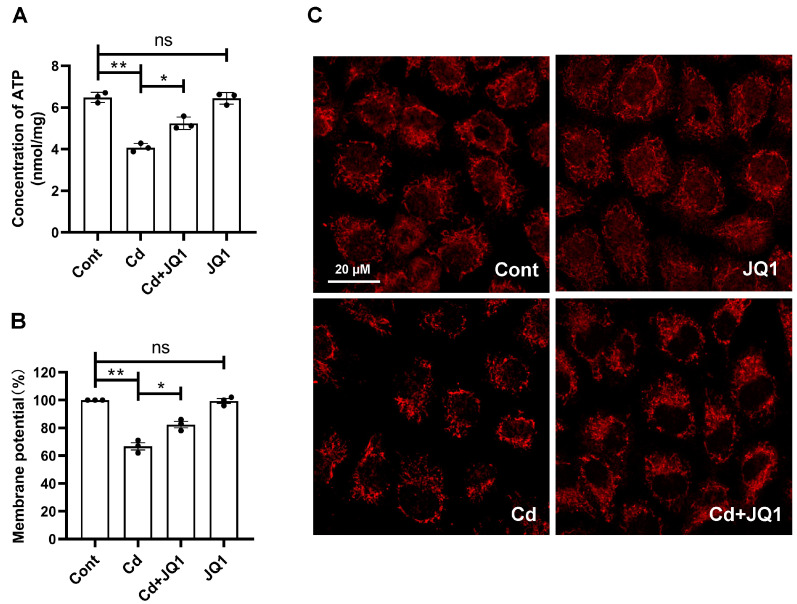
The inhibition of BRD4 ameliorates Cd-induced mitochondrial dysfunction. The concentration of adenosine triphosphate (ATP) (**A**), mitochondrial membrane potential (**B**), and mito-tracker red (MTR) mitochondria morphology (**C**) were detected to evaluate the mitochondrial function. Mean ± SD, n = 3. ns not significant, * p < 0.05, ** p < 0.01.

BRD4 has been found to influence the expression of Nox4, a factor that contributes to mitochondrial dysfunction and the production of ROS. Our findings indicated that JQ1 treatment led to a decrease in Cd-induced Nox4 expression levels (Figure 4A,B). Additionally, the transcriptional levels of Nox4 were measured through qRT-PCR. The results confirmed that JQ1 treatment reduced the transcription levels of Nox4, which were elevated by Cd exposure (Figure 4C). The detection of Nox4 protein expression was conducted via IF staining, where the data presented in Figure 4D demonstrate that JQ1 treatment decreased Cd-induced Nox4 expression levels, aligning with the outcomes of the Western blot analysis. Subsequently, the impact of BRD4 on mitochondrial function was examined in TKPTS cells after Cd exposure. The data illustrated in Figure 5A reveal that JQ1 treatment significantly improved mitochondrial ATP production, which had been inhibited by Cd. Moreover, the results shown in Figure 5B indicate that JQ1 treatment elevated the mitochondrial membrane potential, reduced by Cd. To assess the morphology of the mitochondria, MTR was employed. As depicted in Figure 5C, JQ1 treatment restored the structural integrity of the mitochondria compromised by Cd. In conclusion, inhibiting BRD4 decreases Nox4 expression, restores mitochondrial functionality, and diminishes ROS production in mouse kidney tissues exposed to Cd.

### 3.5. JQ1 Treatment Enhances Nrf2-Mediated Antioxidant Defense System After Cd Exposure

**Figure 6:**
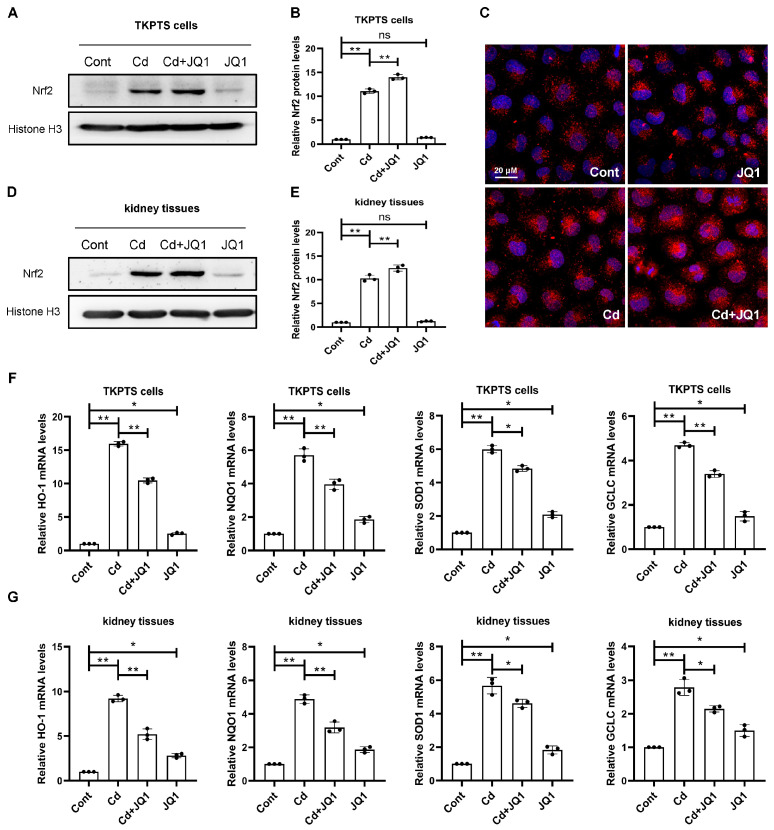
The inhibition of BRD4 promotes the nuclear translocation levels of Nrf2. (**A**) The nuclear translocation levels of Nrf2 were detected by Western blot analysis in TKPTS cells. (**B**) An analysis of the densitometry for Nrf2 nuclear translocation levels was performed. (**C**) IF staining was used to detect the cellular localization of Nrf2. (**D**) The nuclear translocation levels of Nrf2 were detected by Western blot analysis in kidney tissues. (**E**) A densitometry analysis of Nrf2 nuclear translocation levels. (**F**) The transcription levels of HO-1, NQO1, SOD1, and GCLC in TKPTS cells. (**G**) The transcription levels of HO-1, NQO1, SOD1, and GCLC in kidney tissues. Mean ± SD, n = 3. ns not significant, * p < 0.05, ** p < 0.01.

Next, this study aimed to determine whether BRD4 is involved in the regulation of the antioxidant defense system by regulating Nrf2. To investigate the alterations in Nrf2 signaling following treatment with JQ1, the nuclear translocation levels of Nrf2 were measured. The results indicated that JQ1 treatment led to increased nuclear translocation levels of Nrf2, both in vitro (Figure 6A,B) and in vivo (Figure 6D,E). Furthermore, IF staining demonstrated that JQ1 treatment facilitated the nuclear translocation levels of Nrf2 (Figure 6C). Additionally, the transcription levels of antioxidant enzymes regulated by Nrf2 were assessed in both in vitro (Figure 6F) and in vivo (Figure 6G) experiments, revealing that JQ1 treatment enhanced the transcription levels of these enzymes. Collectively, these findings suggest that BRD4 strengthens the antioxidant defense system in kidney cells by promoting the nuclear translocation levels of Nrf2, ultimately mitigating oxidative damage induced by Cd exposure.

## 4. Discussion

Oxidative stress is a prevalent mechanism underlying Cd-induced kidney injury. BRD4 has been shown to play a role in regulating oxidative stress during the onset and progression of various diseases [26]. In this study, the results showed that BRD4 was activated in mice kidneys after Cd exposure, while inhibiting BRD4 mitigated Cd-induced oxidative stress and kidney injury. Additionally, BRD4 inhibition reduced mitochondrial damage and decreased ROS production by suppressing the expression of Nox4 while enhancing the antioxidant defense system through the promotion of nuclear translocation levels of Nrf2. In summary, these findings identify BRD4 as a potential therapeutic target for treating oxidative stress induced by Cd exposure and elucidate the mechanisms through which BRD4 mediates Cd-induced renal oxidative damage.

In normal circumstances, the ROS produced in cellular metabolism regulates numerous important signaling pathways [27]. However, when the balance of the redox system is disrupted, the accumulation of intracellular oxidative products leads to a susceptible state of the organism and then accelerates the occurrence of diseases such as neurodegeneration, inflammation, tumors, and diabetes [28,29]. Exposure to Cd can lead to mitochondrial damage, increase the production of ROS, impair the intracellular antioxidant defense mechanisms, and result in oxidative damage to the kidneys [30,31]. Research has indicated that BRD4 plays a role in the development of various diseases through its regulation of oxidative stress. Zou et al. reported that treatment with the BRD4 inhibitor JQ1 decreases cGAS-STING activation, which, in turn, helps to reduce inflammation and degeneration in the retina caused by oxidative stress in mice [32]. Furthermore, targeted degradation of the BRD4 protein using dBET1 has been shown to improve outcomes in acute ischemic brain injury, with benefits including decreased neuroinflammation, reduced oxidative stress, and the maintenance of blood–brain barrier integrity [33]. Consistent with these findings, the current study revealed that inhibiting BRD4 lessens Cd-induced kidney injury by lowering the oxidative stress level in mice.

The subsequent investigation focused on elucidating the mechanisms through which BRD4 regulates Cd-induced oxidative stress. Nox is a multicomponent enzyme that is widely present in the body and may be a potential activation target of Cd [34]. The Nox family comprises seven enzymes that regulate biological processes such as cellular oxidation–reduction, cell proliferation, and others [35,36]. In human astrocytes, Nox4 inhibits mitochondrial respiration and ATP production, which, in turn, leads to mitochondrial dysfunction and promotes oxidative stress [37]. Mitochondrial dysfunction has been demonstrated to induce the production of mitochondrial and cytoplasmic ROS, thereby exacerbating the vicious cycle of mitochondrial damage and ultimately leading to persistent tissue damage [38,39]. The epigenetic factor BRD4 was identified as a regulator of the transcription and expression of Nox4 [40]. The present study demonstrated that Cd activates Nox4, which may be responsible for mitochondrial damage and ROS generation. Furthermore, the inhibition of Nox4 results in suppression of the repair of mitochondrial function and the oxidative stress level.

The nuclear transcription factor Nrf2 binds to the molecular chaperone Kelch-like ECH-associated protein 1 (Keap1) without stimuli. After stimulation by ROS, Nrf2 dissociates from Keap1 and transports to the nucleus, initiating the transcription and expression of downstream antioxidant enzymes, ultimately protecting cells from oxidative damage [41,42]. The roles of Nrf2 in oxidative stress-induced diseases have been extensively studied. Fang et al. found that astaxanthin promotes the nuclear translocation levels of Nrf2, thereby decreasing the oxidative stress level in retinal tissues of diabetic retinopathy rats [43]. A study also found that the Nrf2 signaling pathway holds significant potential as a therapeutic target in combating oxidative stress associated with corneal diseases [44]. BRD4 has the capacity to competitively bind Keap1, thereby reducing the stability of Nrf2. Furthermore, BRD4 can also directly bind to the Nrf2 protein in a Keap1-independent manner, thereby inhibiting the activity of Nrf2 [45,46]. In this study, the inhibition of BRD4 facilitated the nuclear translocation levels of Nrf2 and recovered the expression of antioxidant proteins, thereby enhancing the antioxidant defense system in renal cells, representing another crucial pathway through which BRD4 regulates the level of oxidative stress in an organism.

With the intensive study of epigenetics, researchers have elucidated the role of BRD4 in Cd-induced kidney injury. Gong et al. discovered that activated BRD4 mediates Cd-induced kidney injury and that BRD4-regulated autophagy inhibition and inflammatory response are important contributors to kidney injury. In contrast, the present study explored the detailed mechanism of BRD4 regulation of oxidative stress. Both studies provide a foundation for targeting BRD4 in the prevention and treatment of cadmium-induced kidney injury [23,47]. Previous studies have addressed the involvement of multiple regulatory factors and pathways in Cd-induced oxidative stress and kidney injury. There is a consensus that Cd-damaged mitochondria lead to increased generation of ROS, accompanied by weakened cellular defense systems, including the Nrf2 pathway, and thus exacerbate the level of oxidative stress [48,49]. On this basis, the present study identified the dual role of the epigenetic factor BRD4 in the regulation of ROS generation and scavenging, further elucidating the potential mechanism of cadmium-induced oxidative stress in the kidney.

## 5. Conclusions

In conclusion, these findings reveal the mechanisms of BRD4 in Cd-induced kidney injury. Cd exposure-activated BRD4 mediates oxidative stress and kidney injury, while JQ1 treatment attenuates ROS generation by regulating Nox4 and enhances ROS scavenging by regulating Nrf2, thereby alleviating Cd-induced kidney oxidative damage. Collectively, these results suggest that BRD4 may serve as a promising therapeutic target for addressing Cd-induced nephrotoxicity.

## Data Availability

The datasets used and/or analyzed during the current study are available from the corresponding author on reasonable request.

## Supplementary Materials

The following supporting information can be downloaded at: https://www.mdpi.com/article/10.3390/toxics13040258/s1, Figure S1: Original images for blots; Table S1: Sequences of primers used in qPCR.
