# Supplementary material for: BRD4 Mediates Cadmium-Induced Oxidative Stress and Kidney Injury in Mice via Disruption of Redox Homeostasis
Source: Toxics. 2025 Mar 29;13(4):258. doi: 10.3390/toxics13040258 (PMC12031608; doi:10.3390/toxics13040258)
Supplement: Supplementary file 1 [file toxics-13-00258-s001.zip › Supplementary Table S1.pdf]

Table S1. Sequences of primers used in qPCR

Target genes	Primer sequences (5'–3')
β-actin	F:TATGCTCTCCCTCACGCCATCC
	R:GTCACGCACGATTTCCCTCTCAG
BRD4	F:CCAAGATGCCTGATGAGCCTGAAG
	R:CTGTCGCTGTCGGAAGAACTGTC
Nox4	F:GCATGTAGCCGCCCACTTGG
	R:AGACACCCGTCAGACCAGGAATG
HO-1	F:ACCGCCTTCCTGCTCAACATTG
	R:CTCTGACGAAGTGACGCCATCTG
NQO1	F:AGAAGCGTCTGGAGACTGTCTGG
	R:GATCTGGTTGTCGGCTGGAATGG
SOD1	F:GACTGCTGGAAAGGACGGTGTG
	R:ACGGCCAATGATGGAATGCTCTC
GCLC	F:AGCTCCTGGAGGAAGGCATCG
	R:GGTCAGACTCGTTGGCATCATCC
