# Supplementary figures and images for: BRD4 Mediates Cadmium-Induced Oxidative Stress and Kidney Injury in Mice via Disruption of Redox Homeostasis
Source: Toxics. 2025 Mar 29;13(4):258. doi: 10.3390/toxics13040258 (PMC12031608; doi:10.3390/toxics13040258)

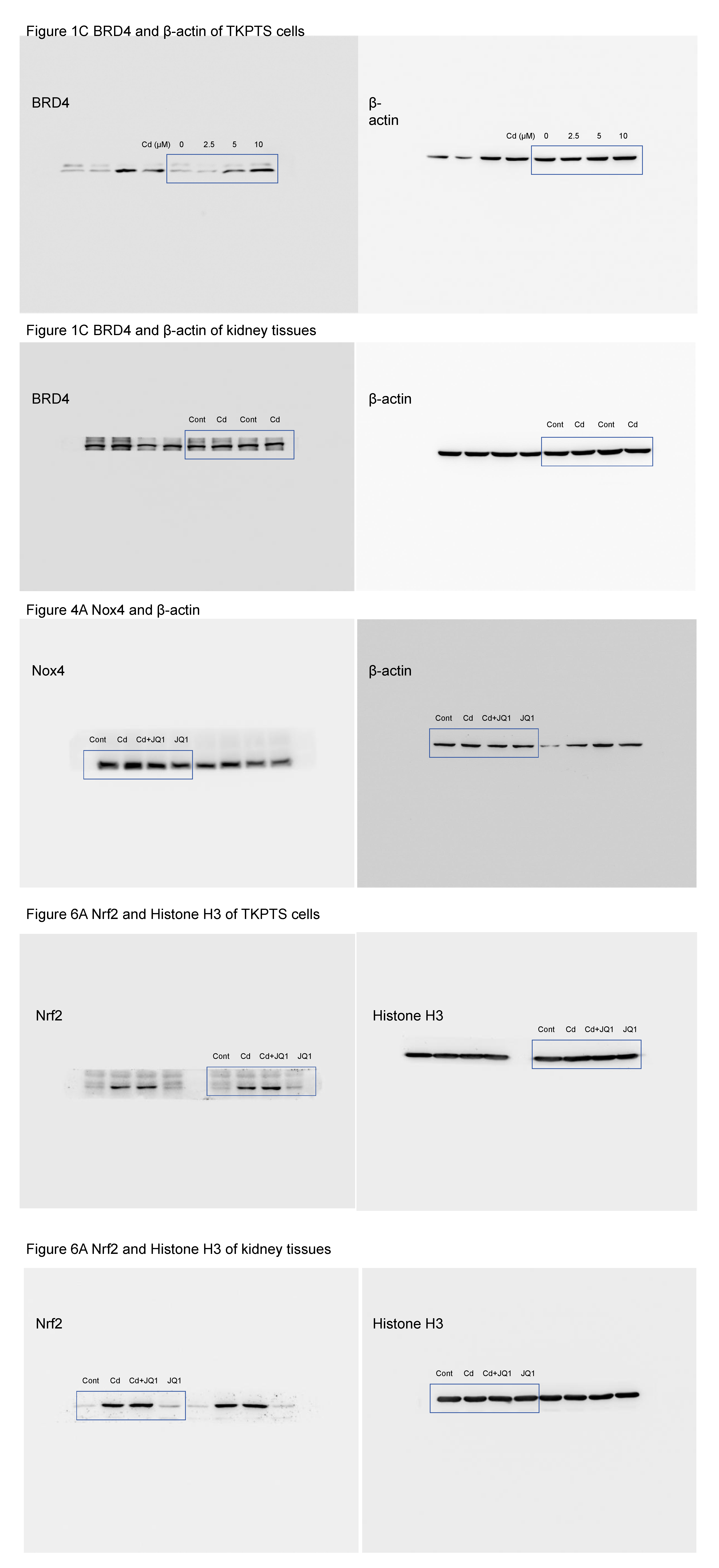

Supplement: Supplementary file 1 [file toxics-13-00258-s001.zip › Supplementary Figure S1.jpg]
